# Identification and characterization of ARID1A-interacting proteins in renal tubular cells and their molecular regulation of angiogenesis

**DOI:** 10.1186/s12967-023-04750-y

**Published:** 2023-11-28

**Authors:** Sunisa Yoodee, Paleerath Peerapen, Sirikanya Plumworasawat, Thanyalak Malaitad, Visith Thongboonkerd

**Affiliations:** Medical Proteomics Unit, Research Department, Faculty of Medicine Siriraj Hospital, Mahidol University, 6thFloor - SiMR Building, 2 Wanglang Road, Bangkoknoi, Bangkok, 10700 Thailand

**Keywords:** Actin, Endothelial cells, Interacting proteins, Renal cancer, Renal epithelial cells, Tumor suppressor

## Abstract

**Background:**

Defects and deficiency of AT-rich interactive domain-containing protein 1A (ARID1A) encoded by a tumor suppressor gene *ARID1A* have recently been suggested to get involved in angiogenesis, a crucial process in carcinogenesis. However, molecular mechanisms of *ARID1A* deficiency to induce angiogenesis in kidney cancer remain underinvestigated.

**Methods:**

We performed large-scale identification of ARID1A protein interactors in renal tubular epithelial cells (RTECs) using immunoprecipitation (IP) followed by nanoLC-ESI-LTQ-Orbitrap tandem mass spectrometry (MS/MS). Their roles in angiogenesis were investigated using various assays.

**Results:**

A total of 74 ARID1A-interacting proteins were identified. Protein–protein interactions analysis revealed that these identified proteins interacted directly or indirectly with ARID1A. Among them, the direct interaction between ARID1A and β-actin was validated by IP and reciprocal IP followed by Western blotting. Small interfering RNA (siRNA) was used for single and double knockdowns of *ARID1A* and *ACTB*. Semi-quantitative RT-PCR demonstrated that deficiency of *ARID1A*, but not *ACTB*, significantly affected expression of angiogenesis-related genes in RTECs (*VEGF* and *FGF2* were increased, whereas *PDGF* and *EGF* were decreased). However, the knockdowns did not affect *TGFB1* and *FGF1* levels. The quantitative mRNA expression data of *VEGF* and *TGFB1* were consistent with the secreted levels of their protein products as measured by ELISA. Only secreted products derived from *ARID1A*-deficient RTECs significantly increased endothelial cells (ECs) migration and tube formation. Some of the other carcinogenic features could also be confirmed in the *ARID1A*-deficient RTECs, including increased cell migration and chemoresistance. Double knockdowns of both *ARID1A* and *ACTB* did not enhance the effects of single *ARID1A* knockdown in all assays.

**Conclusions:**

We report herein a large dataset of the ARID1A-interacting proteins in RTECs using an IP-MS/MS approach and confirm the direct interaction between ARID1A and β-actin. However, the role of ARID1A deficiency in angiogenesis is independent of β-actin.

**Supplementary Information:**

The online version contains supplementary material available at 10.1186/s12967-023-04750-y.

## Introduction

AT-rich interactive domain-containing protein 1A (ARID1A) is a crucial DNA-binding subunit of BAF (BRG1/BRM-associated factor) in the SWI/SNF (SWItch/Sucrose Non-Fermentable) complex [1, 2]. BAF plays role in remodelling chromatin structure by removing or deleting nucleosomes to create the space for transcription factor binding, which is crucial for gene regulation [3, 4]. *ARID1A* gene also serves as a tumor suppressor and is frequently mutated in several cancers, including bladder [5], hepatic [6], colorectal [7] and renal [8] cancers. Besides, the decreased *ARID1A* expression correlates with the poor outcome of cancers [9, 10]. Many cancer reports have shown that ARID1A deficiency impairs the chromatin remodelling complex function, resulting in dysregulation of the carcinogenic gene expression [10–12].

During carcinogenesis, creation of new blood vessels or angiogenesis is one among crucial steps affecting cancer survival and aggressiveness [13, 14]. Mechanistically, cancer cells increase production and secretion of various angiogenic factors, including growth hormones and cytokines, into extracellular matrix (ECM) to activate endothelial cells (ECs) [15, 16]. The activated ECs can degrade endothelial basement membrane to allow them to migrate into the ECM [17]. The migrated ECs then proliferate, further migrate toward the source of stimulants, form the hollow tubes, and finally create new vascular meshes [17]. Recent evidence has demonstrated the involvement of ARID1A in regulating angiogenesis [18, 19]. Silencing *Arid1a* gene in murine hepatocellular carcinoma (HCC) cells increases blood vessel density in the tumor by inducing expression of *Ang2*, a gene encoding angiogenic factor angiopoietin 2 (ANG2) [18]. Similarly, knockdown of *ARID1A* in human ECs activates secretion of ANG2 and promotes ECs proliferation, migration and formation of capillary/mesh-like tubes [19]. Besides, the increase of vascular endothelial growth factor (VEGF), another essential angiogenic factor, has been found in the *ARID1A*-deficient breast [20] and colon [21] cancer cells.

In the kidney, ARID1A deficiency can trigger several carcinogenic features in renal tubular epithelial cells (RTECs), including increased cell proliferation/migration/invasion, spheroid formation, chemoresistance and epithelial-mesenchymal transition (EMT) [22]. However, molecular mechanisms of ARID1A deficiency to regulate angiogenesis in kidney cancer remain underinvestigated. This study thus aimed to define the ARID1A interactors in RTECs and investigate their role in angiogenesis. The ARID1A-interacting proteins in RTECs were isolated and identified by immunoprecipitation (IP) followed by nanoLC-ESI-LTQ-Orbitrap tandem mass spectrometry (MS/MS). Bioinformatics was employed to examine the interactions between ARID1A and its interactors, as well as their functional enrichment. The MS/MS data were then validated by IP and reciprocal IP of the selected ARID1A-interacting partner followed by immunoblotting. For functional analysis, single and double gene knockdowns by small-interfering RNA (siRNA) were performed to suppress mRNA levels of *ARID1A* and its interactor. Expression levels of angiogenesis-related genes, including *VEGF*, *FGF1*, *FGF2*, *PDGF*, *EGF* and *TGFB1*, were measured using semi-quantitative RT-PCR. The secreted levels of VEGF and transforming growth factor-β1 (TGF-β1) proteins were measured by ELISA. Moreover, effects of the secreted products from these siRNA-transfected RTECs on angiogenesis features of ECs were investigated. Finally, some of the other carcinogenic features, including increased cell migration and chemoresistance, were assessed in the siRNA-transfected RTECs.

## Materials and methods

### Cell culture

MDCK cell line (ATCC; Manassas, VA) representing renal tubular epithelial cells (RTECs) and EA.hy926 cell line (ATCC) representing endothelial cells (ECs) were propagated and maintained in a compete medium containing Dulbecco's modified eagle medium (DMEM) (Gibco; Grand Island, NY) supplemented with 10% fetal bovine serum (FBS) (Gibco), 60 U/ml penicillin G (Sigma-Aldrich; St. Louis, MO), and 60 µg/ml streptomycin (Sigma-Aldrich). The cells were cultured in a humidified incubator at 37 ℃ with 5% CO_2_.

### IP and reciprocal IP

Cellular proteins were extracted from MDCK cells using pre-chilled radio-immunoprecipitation assay (RIPA) buffer containing 50 mM Tris–HCl (pH 7.4), 150 mM NaCl, 1 mM EDTA and 0.5% Triton X-100 and a probe sonicator (Sonics Vibra-Cell VCX130) (Sonic & Materials Inc.; Newtown, CT). Cell debris and other remaining particles were removed from the cell lysate by centrifugation at 10,000 ×*g* and 4 °C for 15 min. To reduce non-specific bindings, the clear cell lysate was precleared with 50 µl protein G sepharose beads (50% slurry) (GE Healthcare; Uppsala, Sweden) at 4 °C for 15 min on a tube rotator. After centrifugation at 1500 × *g* and 4 °C for 5 min, the clear supernatant was collected for IP and reciprocal IP as described previously [23, 24].

Briefly, 1 mg pre-cleared lysate (in 1 ml) was incubated with 2 μg mouse monoclonal anti-ARID1A antibody (Santa Cruz Biotechnology; Santa Cruz, CA), mouse monoclonal anti-β-actin antibody (Santa Cruz Biotechnology), or isotype IgG (Santa Cruz Biotechnology) at 4 °C overnight on a tube rotator. The mixture was then incubated with 50 µl protein G sepharose beads (50% slurry) (GE Healthcare) at 4 °C for 4 h on a tube rotator. The beads bound with protein complex were collected by centrifugation at 1500 × *g* and 4 °C for 5 min and washed with RIPA buffer. The immunoprecipitated proteins were then eluted from the beads using 1 × Laemmli’s buffer and resolved by 12% SDS-PAGE. The resolved protein bands were visualized by Oriole fluorescence gel stain (Bio-Rad Laboratories; Hercules, CA) and ChemiDoc MP Imaging System (Bio-Rad Laboratories). These immunoprecipitated proteins were then subjected to MS/MS protein identification, Western blot analysis and other investigations as described below.

### In-gel tryptic digestion and MS/MS protein identification

After excising the resolved bands into multiple gel slices, all the proteins immunoprecipitated by using anti-ARID1A antibody and isotype IgG were subjected to in-gel tryptic digestion [25, 26] and nanoLC-ESI-LTQ-Orbitrap MS/MS protein identification [27, 28] as described previously. More details are also provided in Additional file 2.

### Protein–protein interactions network and functional enrichment analyses

After subtraction of the non-specific binding proteins (identified from the isotype IgG control sample), all the unique proteins identified in the anti-ARID1A-IP sample were summarized and analyzed for their interactions and functional classification using the STRING tool (version 11.5) (https://string-db.org). The confidence level was set at medium (0.40 < score < 0.70).

### Western blotting

Western blot analyses for ARID1A, β-actin and GAPDH (loading control) were performed as described previously [21, 22]. Details are also provided in Additional file 2.

### Single or double gene knockdowns by siRNA in MDCK cells

To address functional roles of ARID1A and its selected interactor, β-actin, siRNA transfection was performed for single and double gene knockdowns in MDCK renal cells. Details of siRNA transfection have been described previously [21, 22] and are also provided in Additional file 2.

### Semi-quantitative RT-PCR

Semi-quantitative RT-PCR was performed as described previously [21, 22]. Briefly, total RNA was isolated from the siRNA-transfected MDCK cells using TRIzol reagent (Thermo Fisher Scientific; Waltham, MA) and Direct-zol RNA MiniPrep (Zymo Research; Irvine, CA). The cDNA synthesis was performed using Viva cDNA Synthesis Kit (Vivantis; Selangor Darul Ehsan, Malaysia) for converting RNA into first-strand cDNA. Semi-quantitative RT-PCR was performed to measure expression levels of *ARID1A*, *ACTB*, *VEGF*, *PDGF*, *EGF*, *TGFB1*, *FGF1* and *FGF2*, whereas *GAPDH* was used to normalize expression levels of these genes. The PCR reaction was done with Taq DNA polymerase (New England Biolabs; Ipswich, MA) and gene-specific primer pairs (Table 1). The thermocycling condition for each PCR reaction was set according to the manufacturer’s guideline with various annealing temperatures (50–60 °C). After 27 cycles of PCR amplification, the PCR fragments were estimated by 1.5% agarose gel electrophoresis, and the gel was stained with ViSafe Red Gel Stain (Vivantis). The DNA bands were detected using ChemiDoc MP Imaging System (Bio-Rad Laboratories), and the band intensities were quantified by using ImageQuant TL software (GE Healthcare).

**Table 1:** Summary of all gene-specific primer pairs used for semi-quantitative RT-PCR

Target gene	Sequence (5ʹ → 3ʹ)		PCR product size
*ACTB*	Forward:	TTTGAGACCTTCAACACCC	209 bp
	Reverse:	AGGATCTTCATGAGGTAGTC	
*ARID1A*	Forward:	CCCCTCAATGACCTCCAGTA	159 bp
	Reverse:	CTGGAAATCCCTGATGTGCT	
*EGF*	Forward:	GAATCATGGCTGTACTCTTG	317 bp
	Reverse:	GGTCATACCCAGGAAAGC	
*FGF1*	Forward:	ACAGTGGATGGGACAAG	287 bp
	Reverse:	TAGTGAGTCCGAGGACC	
*FGF2*	Forward:	CACTTCAAGGACCCCAA	310 bp
	Reverse:	CAGTGCCACATACCAACTG	
*GAPDH*	Forward:	CATCACTGCCACCCAGAAGA	291 bp
	Reverse:	GTGTAGCCCAGGATGCCTTT	
*PDGFA*	Forward:	TGGAGATAGACTCCGTAGG	162 bp
	Reverse:	TGACCGTCCTGGTCTTG	
*TGFβ1*	Forward:	AGTCAAGAAAAGTCCGCACAG	180 bp
	Reverse:	CTGAGGTAGCGCCAGGAATC	
*VEGFA*	Forward:	CGAAGTGGTGAAGTTCATG	240 bp
	Reverse:	CCTATGTGCTGGCCTTG	

### Collection of conditioned medium (CM) from the siRNA-transfected MDCK cells

After the transfection as described above, the siRNA-transfected cells were washed with PBS and incubated in serum-free medium at 37 °C with 5% CO_2_ for 24 h. The CM from each condition was harvested following centrifugation at 2000 ×*g* for 5 min to remove cellular debris and particles, and then used for measuring secreted protein levels and for EA.hy926 treatment in subsequent experiments.

### ELISA

ELISA was performed to measure secreted levels of VEGF and TGF-β1 proteins from the siRNA-transfected RTECs as described previously [22]. Details are also provided in Additional file 2.

### ECs proliferation assay

EA.hy926 cells (2.5 × 10^4^ cells) were seeded in each of 6-well plate (Corning Costar; Cambridge, MA) containing 2 ml complete medium and incubated at 37 °C with 5% CO_2_ overnight. Thereafter, the culture medium was replaced with 2 ml of the mixture (1:1) of complete medium and CM harvested from the siRNA-transfected MDCK cells. The EA.hy926 cells were further incubated for 72 h, and the mixed complete medium/CM was refreshed every day. Total cell number was counted at 24, 48 and 72 h after cultivation in the mixed complete medium/CM by using a hemacytometer and also by flow cytometry (BD Accuri C6 flow cytometer) (BD Biosciences; San Jose, CA).

### ECs migration assay

Transwell migration assay was performed as described previously [29, 30] to examine the migratory response of EA.hy926 ECs to angiogenic factors secreted from MDCK renal cells. Prior to cell seeding, the membrane insert (8-µm-pore size) of the Transwell plate (0.33 cm^2^ culture area/well) (Corning Costar) was pre-coated with matrigel (BD Biosciences) and incubated at 37 °C for 1 h. EA.hy926 cells (1 × 10^5^ in 200 µl serum-free DMEM) were seeded onto the pre-coated membrane insert in upper chamber of each well. Thereafter, 500 µl CM collected from the siRNA-transfected MDCK cells was added into the lower chamber of each well. The cells were allowed to migrate for 24 h at 37 °C with 5% CO_2_, and those remained on the upper surface of the membrane were swapped out, whereas those migrated to the lower surface of the membrane were fixed with 3.7% (v/v) formaldehyde in PBS for 15 min. The cells were then stained with 0.1 μg/ml Hoechst dye (Invitrogen; Carlsbad, CA) at 25 °C for 10 min and observed under Nikon Eclipse 80i fluorescence microscope (Nikon; Tokyo, Japan). Number of the migrated cells was counted from 15 random fields per each sample.

### ECs tube formation assay

ECs tube formation assay [29, 30] was performed to evaluate the ability of EA.hy926 ECs to form capillary/mesh-like tubes on basement membrane matrix in response to the angiogenic factors secreted from MDCK renal cells. Prior to cell seeding, each well of the 96-well plate (Corning Costar) was pre-coated with 50 μl matrigel (BD Biosciences) at 37 °C for 1 h. EA.hy926 cells (5 × 10^4^ cells/well in 100 μl CM harvested from the siRNA-transfected MDCK cells) were seeded into the pre-coated well and incubated at 37 °C with 5% CO_2_ for 24 h. Thereafter, the capillary/mesh-like tubes were imaged by using Nikon Eclipse Ti-S inverted phase-contrast light microscope (Nikon). To quantify the ECs tube formation, numbers of nodes (junctional part) and meshes (hollow part) were measured from 10 random fields per each sample using the angiogenesis analyzer for ImageJ software (https://imagej.nih.gov/ij/).

### RTECs migration assay

RTECs migration assay was performed to confirm the carcinogenic features of the *ARID1A*-deficient RTECs. After siRNA transfection as described above, the transfected MDCK cells (2 × 10^5^ cells in 200 µl serum-free DMEM) were seeded onto the membrane insert (5-µm-pore size) in the upper chamber of each well of the Transwell plate (0.33 cm^2^ culture area/well) (Corning Costar). Each lower chamber was filled with 500 μl of complete medium (with 10% FBS). After 24-h incubation at 37 °C with 5% CO_2_, the migrated cells on the lower surface of the membrane were analyzed and quantified as described in the ECs migration assay.

### RTECs chemoresistance assay

RTECs chemoresistance assay was performed as described previously [22] to confirm the carcinogenic features of the *ARID1A*-deficient RTECs. After siRNA transfection as described above, the transfected MDCK cells were then incubated with 1 µM docetaxel (Hospira, Lake Forest, IL) at 37 °C with 5% CO_2_ for 24 h. Thereafter, the cells were harvested and resuspended in annexin V-binding buffer containing 10 mM 4-(2-hydroxyethyl)-1-piperazineethanesulfonic acid, 140 mM NaCl, and 2.5 mM CaCl_2_∙2H_2_O (pH 7.4). The cells were then incubated with fluorescein isothiocyanate (FITC)-labeled annexin V for 15 min at 25 °C in the dark and then with 0.2 µg/ml propidium iodide for 5 min at 25 °C in the dark. Finally, cell death was quantified by flow cytometry using BD Accuri C6 flow cytometer (BD Biosciences).

### Statistical analysis

Quantitative data in all experiments were derived from three independent experiments using different sets of biological samples and are shown as mean ± SD. Statistically significant differences were determined using one-way analysis of variance (ANOVA) with Tukey’s post-hoc test or Kruskal–Wallis test (based on data distribution). Statistical significance was indicated by *p*-value < 0.05.

## Results

### IP-MS/MS identification of ARID1A-interacting proteins

**Fig. 1:**
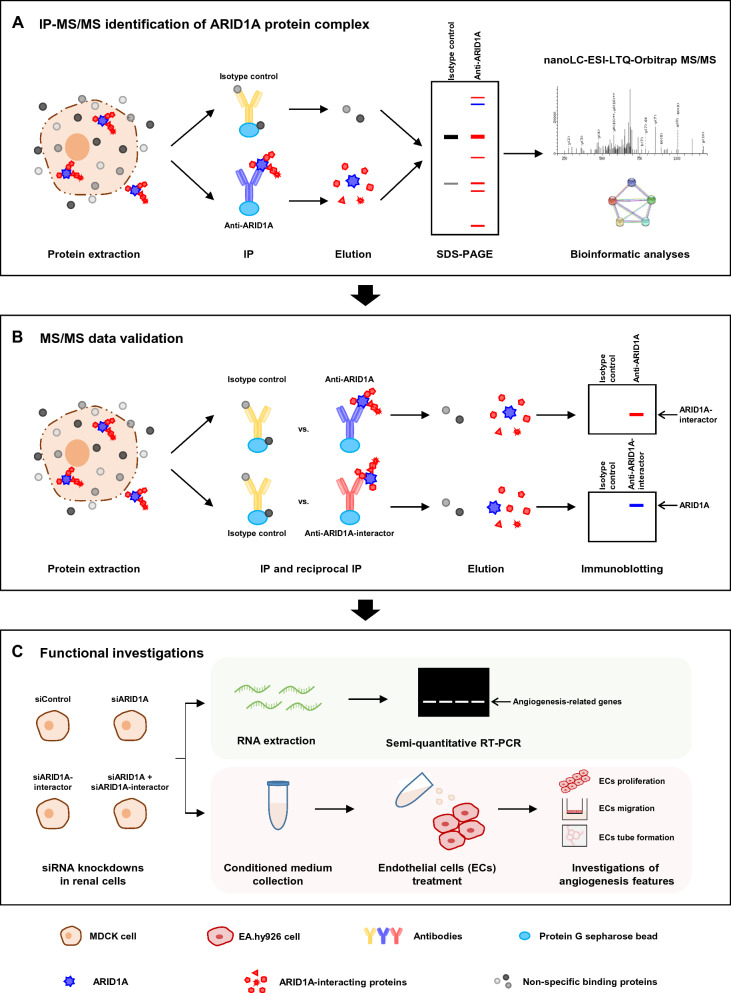
Schematic diagram for analytical methods in this study. **A** ARID1A protein complex in MDCK renal cells was isolated from the whole cell lysate by IP with anti-ARID1A antibody. In parallel, the sample immunoprecipitated with isotype IgG served as the control for background (non-specific) subtraction. The immunoprecipitated proteins were resolved by SDS-PAGE and subjected to in-gel tryptic digestion followed by identification by nanoLC-ESI-LTQ-Orbitrap MS/MS and bioinformatic analyses to predict protein–protein interactions network and functional enrichment. **B** The MS/MS data were validated by IP and reciprocal IP followed by immunoblotting. **C** To study functions, single and double knockdowns of *ARID1A* and its interactor by siRNA were performed in MDCK cells. RNA was extracted and expression levels of angiogenesis-related genes were determined by semi-quantitative RT-PCR. Finally, effects of secreted products derived from the siRNA-transfected MDCK cells on angiogenesis features (including cell proliferation, migration and tube formation) of EA.hy926 ECs were examined

**Fig. 2:**
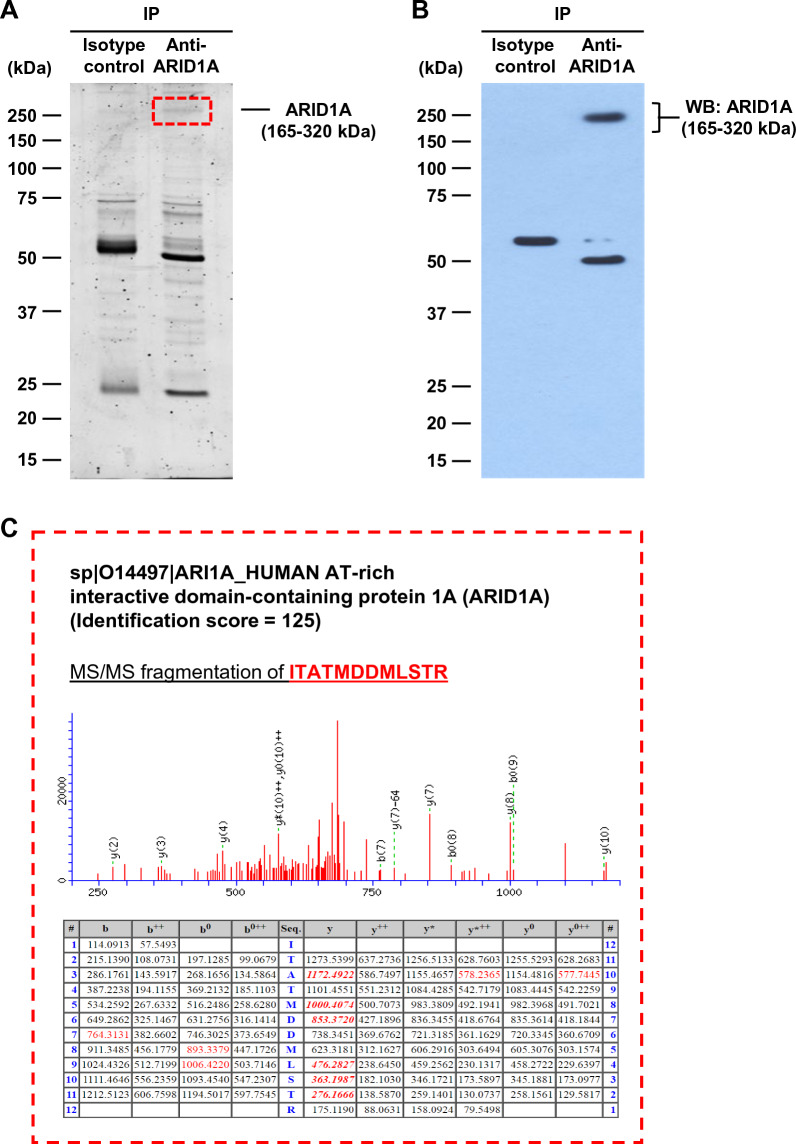
IP-MS/MS identification of ARID1A-interacting proteins. **A** Cellular proteins immunoprecipitated by isotype IgG or anti-ARID1A antibody were resolved by SDS-PAGE and visualized with Oriole fluorescence gel stain. **B** Immunoblotting revealed a distinct ARID1A protein band at approximately 250 kDa in the anti-ARID1A-IP sample. **C** nanoLC-ESI-LTQ-Orbitrap MS/MS analysis confirmed that the 250 kDa-immunoreactive band was ARID1A

The methodology performed in this study is schematically summarized in Fig. 1. IP was performed to pull down the ARID1A protein complex. SDS-PAGE followed by Oriole fluorescence gel stain revealed differential patterns of protein bands resolved from the IP samples using anti-ARID1A antibody versus isotype IgG (Fig. 2A). In particular, a distinct band at approximately 250 kDa was observed only in the anti-ARID1A-IP sample. Immunoblotting confirmed that such distinct band really was the ARID1A protein (Fig. 2B), indicating that ARID1A was successfully pulled down by IP. Subsequently, the SDS-PAGE gel containing the immunoprecipitated proteins was excised into multiple slices and subjected to protein identification by in-gel tryptic digestion followed by nanoLC-ESI-LTQ-Orbitrap MS/MS analyses. As expected, the immunoreactive band at ~ 250 kDa was identified as ARID1A protein (Fig. 2C). After subtraction of some proteins identified from the isotype IgG-IP samples, 74 unique proteins identified exclusively in the anti-ARID1A-IP samples (the ARID1A-interacting proteins) were summarized (Table 2).

**Table 2:** Summary of all 74 unique ARID1A-interacting proteins identified by IP-MS/MS

Protein name	Swissprot ID	Gene symbol	MS/MS identification score	%Cov	No. of unique/total matched peptides	MW (kDa)
14–3-3 protein zeta/delta	P63101	*YWHAZ*	20.18	9.0	2/2	27.77
40S ribosomal protein S12	Q76I81	*RPS12*	33.54	16.7	1/1	14.52
40S ribosomal protein S18	Q5TJE9	*RPS18*	6.18	5.3	1/1	17.72
40Sribosomal protein SA	Q4GWZ2	*RPSA*	34.73	9.8	2/2	32.93
60S ribosomal protein L3	Q4R5Q0	*RPL3*	8.75	2.2	1/1	46.05
60S ribosomal protein L31	Q5RBR9	*RPL31*	6.35	11.2	1/1	14.39
60Sacidic ribosomal protein P0	P19945	*RPLP0*	14.92	6.3	2/2	34.22
Actin, alpha cardiac muscle 1	P68032	*ACTC1*	6.24	22.5	1/9	42.02
Actin, cytoplasmic 1	P84336	*ACTB*	323.31	53.9	1/16	41.80
Actin, cytoplasmic 2	P63261	*ACTG1*	39.50	53.9	1/16	41.79
Alpha-enolase (Fragment)	P86210	*ENO1*	8.29	9.9	1/1	23.84
Caspase-14	P31944	*CASP14*	14.07	10.7	2/2	27.68
Cathepsin D	P07339	*CTSD*	13.92	6.1	2/2	44.55
Cleavage and polyadenylation specificity factor subunit 5	Q9CQF3	*NUDT21*	82.14	20.7	3/3	26.24
Cleavage and polyadenylation specificity factor subunit 7	Q5XI29	*CPSF7*	83.31	10.4	4/4	51.07
Coiled-coil domain-containing protein 39	E2R1I5	*CCDC39*	6.20	0.9	1/1	110.37
Cornifin-B	P22528	*SPRR1B*	34.12	55.1	4/4	9.89
D-3-phosphoglycerate dehydrogenase	Q61753	*PHGDH*	7.86	2.1	1/1	56.59
DCC-interacting protein 13-alpha	Q8K3H0	*APPL1*	33.13	5.0	3/3	79.33
Desmoplakin	P15924	*DSP*	21.59	1.0	2/2	331.77
Dolichyl-diphosphooligosaccharide protein glycosyltransferase 48 kDa subunit	Q29381	*DDOST*	28.26	2.5	1/1	48.86
Dolichyl-diphosphooligosaccharide protein glycosyltransferase subunit 1	E2RQ08	*RPN1*	8.10	2.5	1/1	68.58
Dystrophin	P11532	*DMD*	6.02	0.3	1/1	426.74
Elongation factor 1-delta	Q717R8	*EEF1D*	6.59	3.2	1/1	30.82
Elongation factor 1-gamma (Fragment)	Q29387	*EEF1G*	26.42	3.0	1/1	49.62
Eukaryotic initiation factor 4A-I (Fragment)	P29562	*EIF4A1*	10.27	2.5	1/1	45.29
Filamin-B	Q80X90	*FLNB*	6.91	0.4	1/1	277.82
Fructose-bisphosphate aldolase A	Q5NVR5	*ALDOA*	14.37	11.5	2/2	39.45
Heterogeneous nuclear ribonucleoprotein C	G3V9R8	*HNRNPC*	6.05	3.7	1/1	32.86
Heterogeneous nuclear ribonucleoprotein D0	Q60668	*HNRNPD*	13.29	5.1	2/2	38.35
Heterogeneous nuclear ribonucleoprotein H	Q8VHV7	*HNRNPH1*	20.84	3.8	1/1	49.19
Heterogeneous nuclear ribonucleoprotein K	P61980	*HNRNPK*	25.88	4.3	2/2	50.98
Histone H2A type 1-D	C0HKE3	*H2AC7*	15.46	21.5	2/2	14.14
Ig gamma-1 chain C region secreted form	P01868	*IGHG1*	106.40	27.5	7/7	35.70
Integrin-linked kinase-associated serine/threonine phosphatase 2C	Q0IIF0	*ILKAP*	17.46	7.8	3/3	40.62
Interferon-induced very large GTPase 1	Q80SU7	*GVIN1*	6.44	0.4	1/1	280.81
Interleukin enhancer-binding factor 2	Q9CXY6	*ILF2*	14.77	6.2	2/2	43.06
Myosin-11 (Fragment)	Q63862	*MYH11*	93.09	1.2	1/1	152.49
Olfactory receptor 143	P34985	*OLFR143*	6.31	2.9	1/1	35.20
Poly(rC)-binding protein 2	Q61990	*PCBP2*	7.13	3.6	1/1	38.22
Prohibitin-2	Q99623	*PHB2*	8.03	3.3	1/1	33.30
Prostaglandin reductase 1	Q9EQZ5	*PTGR1*	6.56	3.0	1/1	35.73
Protein disulfide-isomerase	P07237	*P4HB*	6.48	5.9	1/1	57.12
Protein disulfide-isomerase A3	Q5RDG4	*PDIA3*	20.51	2.2	1/1	56.78
Protein S100 − A7	P31151	*S100A7*	105.09	47.5	6/6	11.47
Protein S100−A8	P05109	*S100A8*	99.03	31.2	3/3	10.83
Protein S100−A9	P06702	*S100A9*	124.31	51.8	6/6	13.24
Purine nucl−oside phosphorylase	P85973	*PNP*	6.34	3.5	1/1	32.30
Putative deoxyribonuclease TATDN2	Q93075	*TATDN2*	6.02	1.2	1/1	85.02
Putative heat shock protein HSP 90-beta 2	Q58FF8	*HSP90AB2P*	13.40	7.1	1/2	44.35
Pyruvate kinase PKM	P11980	*PKM*	9.32	4.3	1/1	57.82
RabGDP dissociation inhibitor beta	Q6Q7J2	*GDI2*	6.77	2.5	1/1	50.27
Ras-related protein Rab-11A	Q5R9M7	*RAB11A*	10.52	6.0	1/1	24.39
Ras-related protein Rab-6A	Q9WVB1	*RAB6A*	14.24	5.3	1/1	23.59
RNA binding motif protein, X-linked-like-1	Q91VM5	*RBMXL1*	10.20	3.4	1/1	42.16
RNA-binding protein EWS	Q61545	*EWSR1*	14.89	2.1	1/1	68.46
Semenogelin-1	P04279	*SEMG1*	21.73	7.4	3/3	52.13
Semenogelin-2	Q02383	*SEMG2*	109.43	7.2	2/2	65.44
Serpin B12	Q96P63	*SERPINB12*	6.32	2.0	1/1	46.28
Serpin B3	P29508	*SERPINB3*	26.25	9.2	3/3	44.56
Small proline-rich protein 2D	P22532	*SPRR2D*	6.14	61.1	1/4	7.91
Small proline-rich protein 2E	P22531	*SPRR2E*	36.24	79.2	1/4	7.86
Spectrin beta chain, non-erythrocytic 1	Q62261	*SPTBN1*	6.62	0.4	1/1	274.22
SWI/SNF complex subunit SMARCC2	Q6PDG5	*SMARCC2*	31.10	2.7	2/2	132.60
T-complex protein 1 subunit gamma	Q6P502	*CCT3*	15.91	4.0	2/2	60.65
Transcription activator BRG1	A7Z019	*SMARCA4*	16.83	1.5	2/2	180.68
Transferrin receptor protein 1	Q99376	*TFRC*	6.19	1.2	1/1	85.88
Transitional endoplasmic reticulum ATPase	Q3ZBT1	*VCP*	9.18	1.6	1/1	89.33
Tubulin alpha-1B chain	Q4R538	*TUBA1B*	149.28	18.0	6/6	50.15
Tubulin beta chain	P07437	*TUBB*	19.24	25.9	1/10	49.67
Tubulin beta-2B chain	Q9CWF2	*TUBB2B*	8.52	19.8	1/8	49.95
Tubulin beta-4B chain	Q6P9T8	*TUBB4B*	145.71	29.7	2/11	49.80
Ubiquitin thioesterase OTUB2	Q96DC9	*OTUB2*	9.63	3.0	1/1	27.21
Voltage-dependent anion-selective channel protein 1	Q9Z2L0	*VDAC1*	19.45	3.5	1/1	30.76

%Cov = percentage of sequence coverage

### Protein–protein interactions network and functional enrichment analyses of ARID1A-interactors

**Fig. 3:**
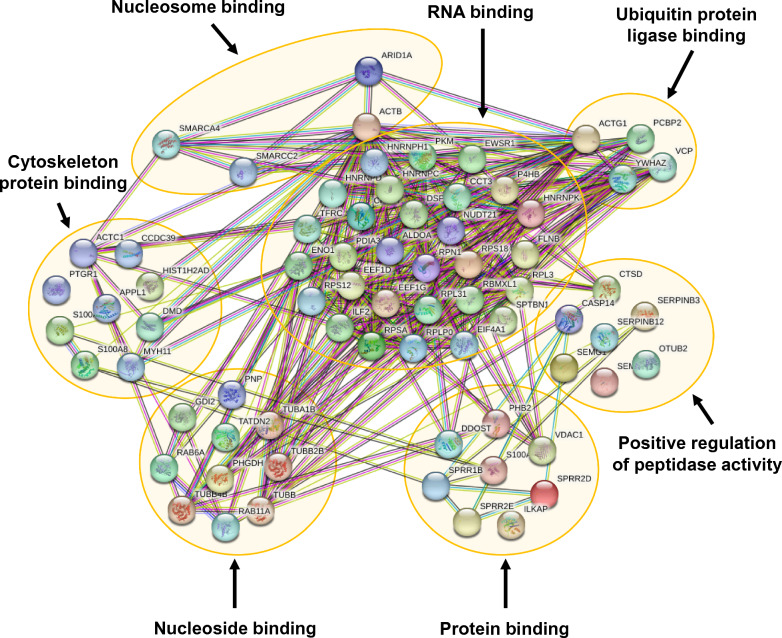
Protein–protein interactions network and functional enrichment analyses of ARID1A-interactors. The interactions and functions of ARID1A and its 74 interactors identified by nanoLC-ESI-LTQ-Orbitrap MS/MS analyses were determined by using the STRING tool (version 11.5) (https://string-db.org) with a medium confidence level (0.40 < score < 0.70)

ARID1A and all 74 interactors were submitted to STRING tool for protein–protein interactions network and functional enrichment analyses. As illustrated in Fig. 3, these identified ARID1A-interacting proteins had either direct or indirect interactions with ARID1A. In addition, ARID1A and its interactors played several functional roles in nucleosome binding, RNA binding, ubiquitin protein ligase binding, cytoskeleton protein binding, nucleoside binding, protein binding, and positive regulation of peptidase activity (Fig. 3).

### Validation of the association between ARID1A and its interactor

**Fig. 4:**
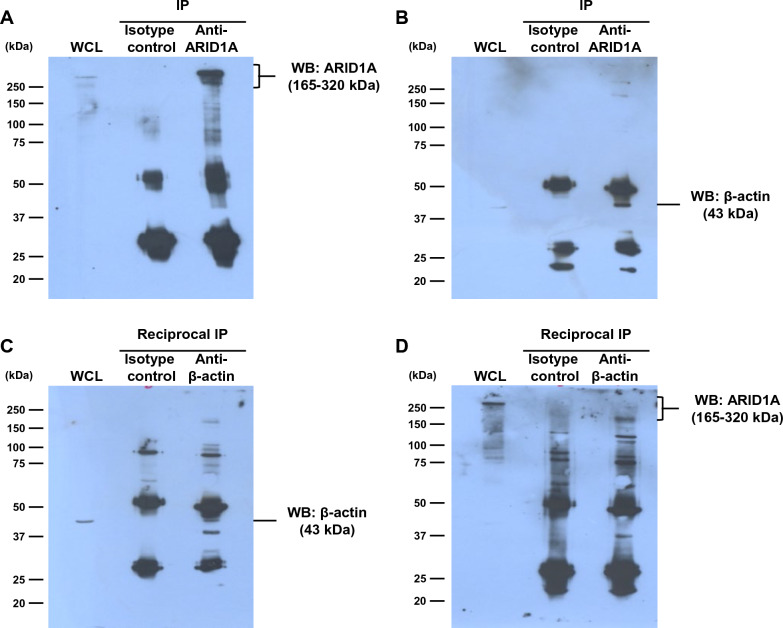
Validation of the association between ARID1A and its interactor. Cellular proteins from MDCK cells were immunoprecipitated with anti-ARID1A (IP) **A**, **B** or anti-β-actin (reciprocal IP) **C**, **D**, whereas those parallelly immunoprecipitated with isotype IgG served as the control for all samples. The immunoprecipitated proteins were then resolved by SDS-PAGE and subjected to immunoblotting to detect ARID1A or β-actin. WCL = whole cell lysate

IP and reciprocal IP followed by immunoblotting were applied for validating the direct association between ARID1A and β-actin (actin, cytoplasmic 1) (encoded by *ACTB*). These combined approaches successfully confirmed the direct association between ARID1A and β-actin. The immunoblots clearly showed that ARID1A and β-actin were detected in both of the anti-ARID1A-IP and anti-β-actin-IP samples, but not in the isotype IgG-IP samples (Fig. 4).

### Single and double knockdowns of *ARID1A* and *ACTB* by siRNA in MDCK renal cells

**Fig. 5:**
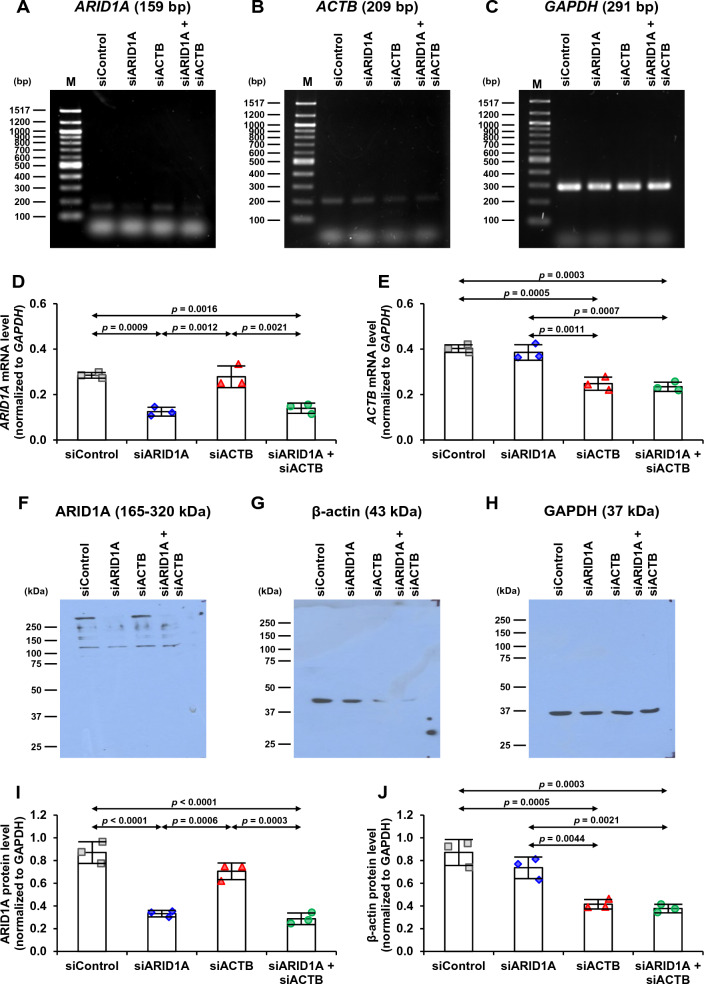
Single and double knockdowns of *ARID1A* and *ACTB* by siRNA in MDCK renal cells. MDCK cells were individually or dually transfected with siRNA specific to *ARID1A* (siARID1A) and *ACTB* (siACTB). Efficacy of the siRNA transfection to silence expression of *ARID1A* and/or *ACTB* was determined by semi-quantitative RT-PCR compared with transfection with the control siRNA (siControl). **A–C** Gel images of mRNA bands of *ARID1A*, *ACTB* and *GAPGH*, respectively. **D–E** Quantitative analysis of mRNA band intensities of *ARID1A* and *ACTB*, respectively, normalized to *GADPH*. **F-H** Immunoblotting of ARID1A, β-actin and GAPDH, respectively. **I–J** Quantitative analysis of protein band intensities of ARID1A and β-actin, respectively, normalized to GADPH. The dots on top of each bar represent individual data points derived from three biological replicates in three independent experiments, whereas the error bar represents mean ± SD of each group. Only significant *p* values are labeled. M = marker ladder

For functional study of ARID1A and its interactors, gene silencing by siRNA specific for ARID1A (siARID1A) and β-actin (siACTB) was performed to decrease expression levels of *ARID1A* and/or *ACTB* in MDCK renal cells. After single and double knockdowns, mRNA levels were confirmed by semi-quantitative RT-PCR. The agarose gel images showed obvious decreases in *ARID1A* (Fig. 5A) and *ACTB* (Fig. 5B) band intensities in the siRNA-transfected cells, whereas that of *GAPDH* remained unchanged and thus served as the house-keeping gene for normalization (Fig. 5C). Quantitative analysis revealed that relative *ARID1A* mRNA level significantly decreased in both siARID1A- and siARID1A + siACTB-transfected cells compared with the cells transfected with control siRNA (siControl) (Fig. 5D). Similarly, the *ACTB* mRNA level significantly decreased in both siACTB- and siARID1A + siACTB-transfected cells compared with siControl-transfected cells (Fig. 5E). Note that double knockdowns did not make further decrease of either *ARID1A* or *ACTB* mRNA level as compared with the single knockdown.

Additionally, protein levels of ARID1A and β-actin were measured by immunoblotting. The immunoblots demonstrated thinner and fainter bands of ARID1A and β-actin in MDCK cells with single or double gene knockdowns (Fig. 5F, G), whereas GAPDH band remained unchanged and thus served as the loading control (Fig. 5H). Quantitative analysis revealed that relative ARID1A protein level significantly decreased in both siARID1A- and siARID1A + siACTB-transfected cells compared with siControl-transfected cells (Fig. 5I). Similarly, the β-actin protein level significantly decreased in both siACTB- and siARID1A + siACTB-transfected cells compared with siControl-transfected cells (Fig. 5J). Note that double knockdowns did not make further decrease of either ARID1A or β-actin protein level as compared with the single knockdown.

### Effects of single and double knockdowns of *ARID1A* and *ACTB* on expression of angiogenesis-related genes and secretion of angiogenic factors in MDCK renal cells

**Fig. 6:**
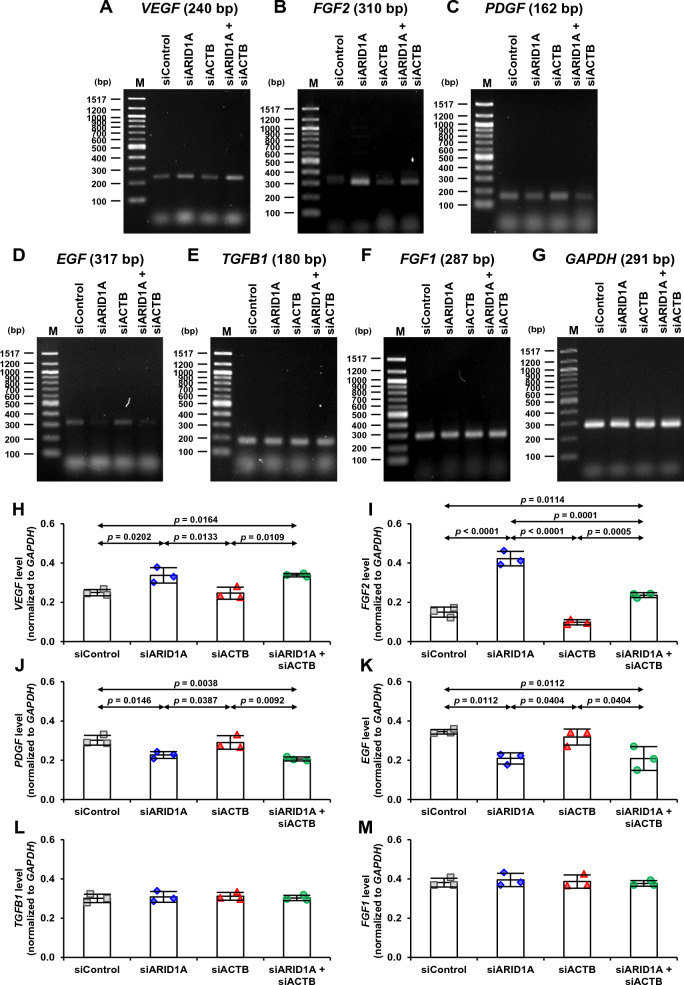
Effects of single and double knockdowns of *ARID1A* and *ACTB* on expression of angiogenesis-related genes in MDCK renal cells. Semi-quantitative RT-PCR was performed to determine expression levels of angiogenesis-related genes in the siRNA-transfected MDCK cells. **A–G** Gel images of mRNA bands of *VEGF*, *FGF2*, *PDGF*, *EGF*, *TGFB1*, *FGF1* and *GAPDH*, respectively. **H–M** Quantitative analysis of mRNA band intensities of *VEGF*, *FGF2*, *PDGF*, *EGF*, *TGFB1* and *FGF1*, respectively, normalized to *GAPDH*. The dots on top of each bar represent individual data points derived from three biological replicates in three independent experiments, whereas the error bar represents mean ± SD of each group. Only significant *p* values are labeled. M = marker ladder

Semi-quantitative RT-PCR was also used to evaluate expression levels of angiogenesis-related genes, including *VEGF*, *FGF2*, *PDGF*, *EGF*, *TGFB1* and *FGF1*, after single and double knockdowns of *ARID1A* and *ACTB* in MDCK cells, whereas *GAPDH* served as a housekeeping gene for normalization (Fig. 6A–G). Quantitative analysis revealed that *VEGF* and *FGF2* significantly increased in the siARID1A- and siARID1A + siACTB-transfected cells, but remained unchanged in the siACTB-transfected cells compared with the siControl-transfected cells (Fig. 6H, I). By contrast, *PDGF* and *EGF* levels significantly decreased in the siARID1A- and siARID1A + siACTB-transfected cells, but remained unchanged in the siACTB-transfected cells (Fig. 6J, K). However, single and double knockdowns of *ARID1A* and *ACTB* had no effects on levels of *TGFB1* and *FGF1* (Fig. 6L, M).

ELISA was performed to confirm that the mRNA expression of these angiogenesis-related genes affected the secreted levels of their protein products. VEGF served as the representative protein with up-regulation induced by *ARID1A* knockdown, whereas TGF-β1 served as the representative unaffected protein. In consistent with the mRNA level, siARID1A- and siARID1A + siACTB-transfected cells had significantly increased level of secreted VEGF, while siACTB-transfection did not affect the secreted VEGF level as compared with the siControl-transfected cells (Additional file 1: Figure S1A). In addition, ELISA revealed that the secreted level of TGF-β1 was not altered by single and double knockdowns of *ARID1A* and *ACTB* (Additional file 1: Figure: S1B). These data confirmed that expression of the angiogenesis-related genes really affected the secretion of their protein products.

### Effects of secreted products derived from the siRNA-transfected MDCK cells on ECs proliferation

**Fig. 7:**
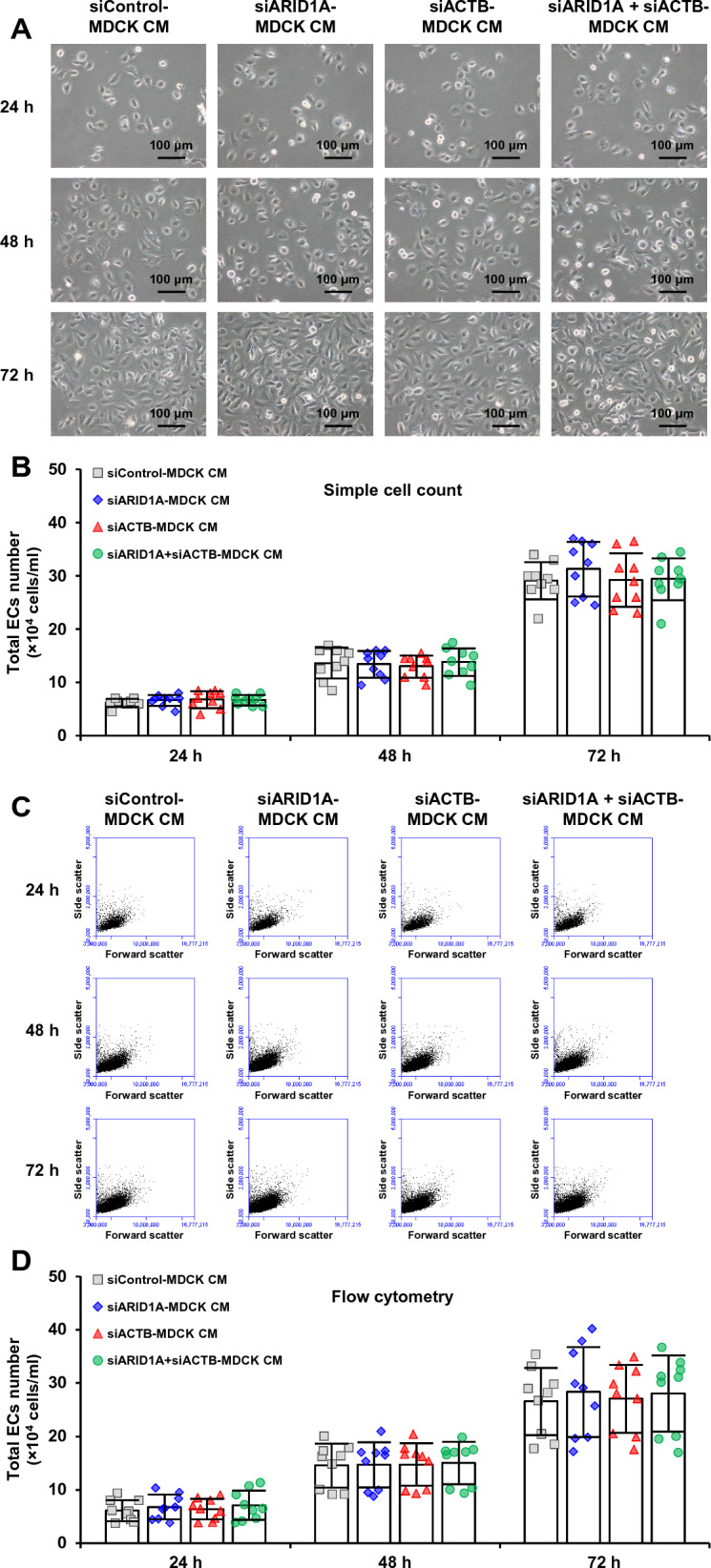
Effects of secreted products derived from the siRNA-transfected MDCK cells on ECs proliferation. Conditioned medium (CM) was harvested after 24-incubation of the siRNA-transfected MDCK cells in serum-free medium. ECs proliferation was determined by incubating EA.hy926 cells in CM mixed (1:1) with complete medium for up to 72 h. The EA.hy926 cells were collected at 24-, 48-, and 72-h time-points and subjected to cell count. **A** Micrographs of EA.hy926 cells after incubation with CM at indicated time-points. **B** Total cell numbers were counted using a hemacytometer. **C** Scatter plots of flow cytometric analysis at indicated time-points. **D** Total cell number quantified by flow cytometry. The dots on top of each bar represent individual data points derived from three biological replicates in three independent experiments, whereas the error bar represents mean ± SD of each group. Only significant *p* values are labeled

To investigate the effects of ARID1A and its interactor in RTECs on angiogenesis, ECs proliferation assay was performed following incubation of ECs with conditioned medium (CM) harvested from the siRNA-transfected MDCK cells. Figure 7A demonstrates morphology of EA.hy926 cells after 24-, 48- and 72-h incubation in CM from the siControl-, siARID1A-, siACTB-, and siARID1A + siACTB-transfected MDCK cells. Simple cell count using hemacytometer revealed that the EA.hy926 cells incubated with CM from MDCK cells transfected with siControl, siARID1A, siACTB, and siARID1A + siACTB had comparable cell numbers at all time-points (Fig. 7B). In consistent, flow cytometry also revealed no significant difference of the cell numbers in all of these EA.hy926 cells incubated with CM from different siRNA-transfected cells at all time-points (Fig. 7C, D).

### Effects of secreted products derived from the siRNA-transfected MDCK cells on ECs migration

**Fig. 8:**
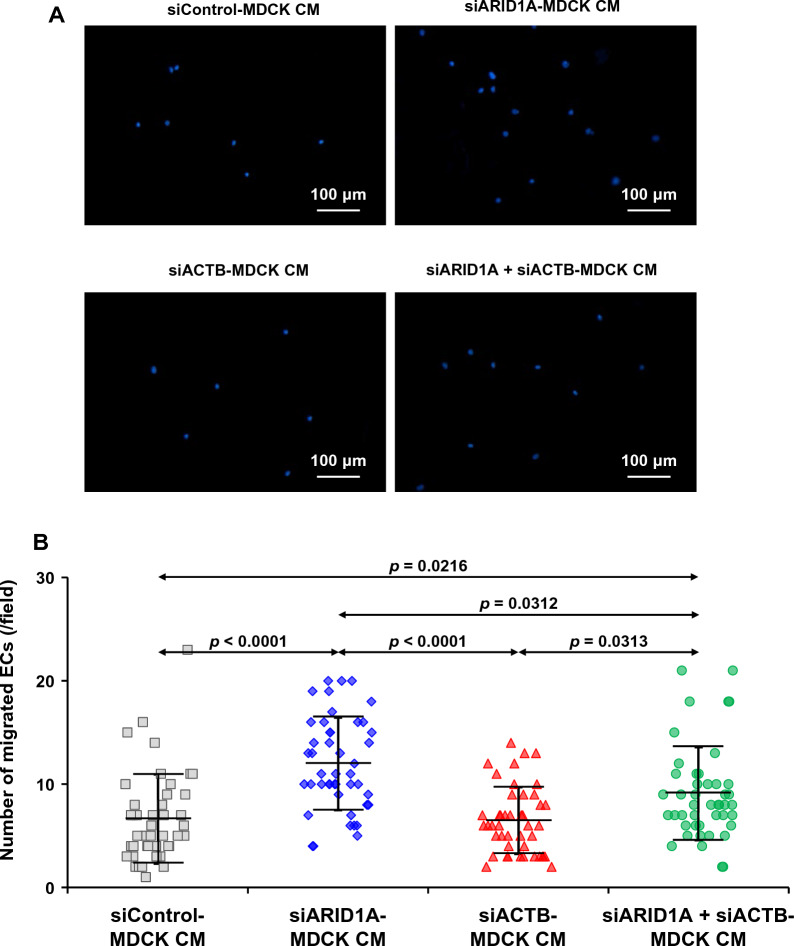
Effects of secreted products derived from the siRNA-transfected MDCK cells on ECs migration. Conditioned medium (CM) was harvested after 24-incubation of the siRNA-transfected MDCK cells in serum-free medium. ECs migration was determined by allowing EA.hy926 cells in the upper chamber with serum-free medium to migrate into in the lower chamber containing CM from each condition. After 24h incubation, the migrated ECs were stained with a fluorescence dye and imaged under the fluorescence microscope. **A** Micrographs of the immunofluorescence-stained migrated ECs in each condition. **B** Numbers of the migrated ECs were counted from 15 random fields per each sample. The dots represent individual data points derived from three biological replicates in three independent experiments, whereas the error bar represents mean ± SD of each group. Only significant *p* values are labeled

The migratory activity of EA.hy926 cells in response to the secreted products from the siRNA-transfected MDCK cells was evaluated using the ECs migration assay. After 24-h incubation with CM from the siRNA-transfected MDCK cells, the migrated cells were stained with a fluorescence dye and imaged under the fluorescence microscope (Fig. 8A). Quantitative analysis revealed that number of the migrated cells was significantly increased by CM derived from the siARID1A- and siARID1A + siACTB-transfected MDCK cells as compared with the EA.hy926 cells incubated with CM from the siControl-transfected MDCK cells (Fig. 8B). However, there was no significant change observed in the EA.hy926 cells incubated with CM derived from the siACTB-transfected MDCK cells (Fig. 8B).

### Effects of secreted products derived from the siRNA-transfected MDCK cells on ECs tube formation

**Fig. 9:**
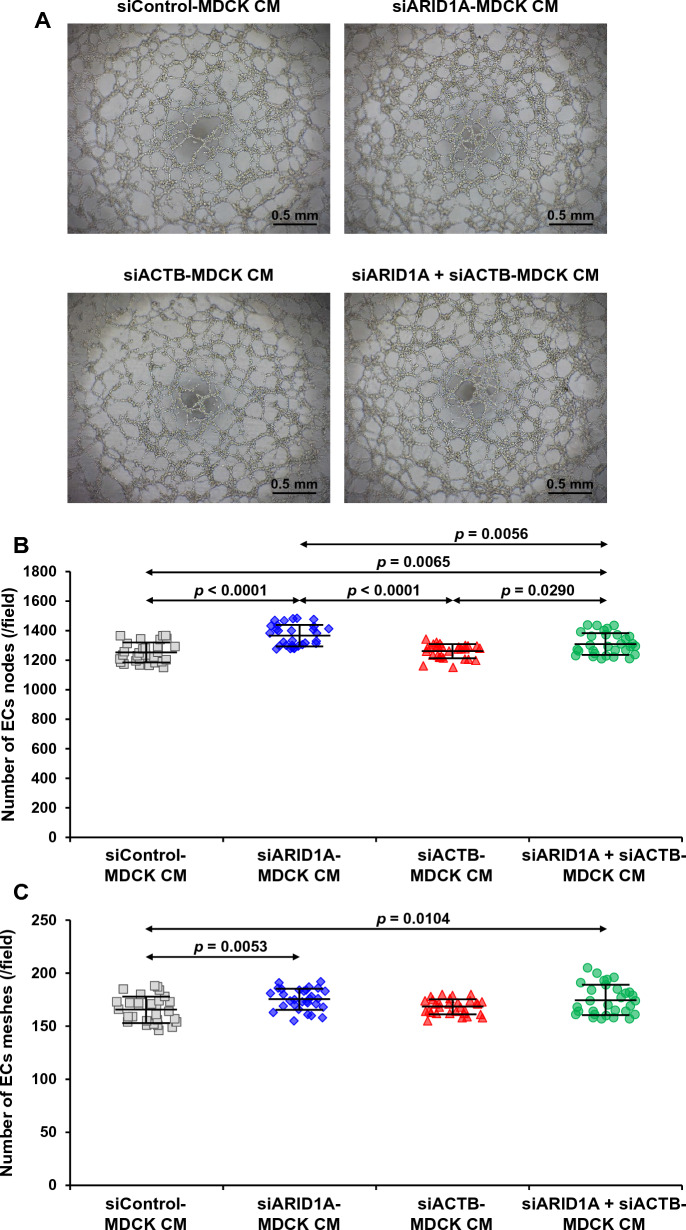
Effects of secreted products derived from the siRNA-transfected MDCK cells on ECs tube formation. Conditioned medium (CM) was harvested after 24-incubation of the siRNA-transfected MDCK cells in serum-free medium. ECs tube formation was determined by incubating EA.hy926 cells for 24 h in matrigel-coated wells containing CM derived from the siRNA-transfected MDCK cells. **A** Micrographs of the capillary/mesh-like tubes in each condition taken by an inverted phase-contrast light microscope. **B–C** Numbers of nodes (junctional part) and meshes (hollow part) of the ECs tubes were measured from 10 random fields per each sample using the angiogenesis analyzer for ImageJ software (https://imagej.nih.gov/ij/). The dots represent individual data points derived from three biological replicates in three independent experiments, whereas the error bar represents mean ± SD of each group. Only significant *p* values are labeled

Ability of EA.hy926 cells to construct capillary/mesh-like tubes in response to the secreted products from the siRNA-transfected MDCK cells was evaluated using the ECs tube formation assay. After 24-h incubation with CM derived from the siRNA-transfected MDCK cells, numbers of ECs tube nodes (junctional part) and meshes (hollow part) were measured. Figure 9A demonstrates the capillary/mesh-like tubes formed by EA.hy926 cells incubated with the secreted products from the siRNA-transfected MDCK cells. Using the angiogenesis analyzer, numbers of nodes and meshes significantly increased in the EA.hy926 cells incubated with CM derived from the siARID1A- and siARID1A + siACTB-transfected MDCK cells as compared with ECs incubated with CM from the siControl-transfected MDCK cells (Fig. 9B, C). However, there was no significant change observed in the EA.hy926 cells incubated with CM derived from the siACTB-transfected MDCK cells (Fig. 9B, C).

### Effects of single and double knockdowns of *ARID1A *and *ACTB* on RTECs migration

To confirm the carcinogenic features of the *ARID1A*-deficient RTECs, the migratory capability of MDCK cells was examined using the migration assay after single and double knockdowns of *ARID1A* and *ACTB*. After 24-h incubation in the Transwell chamber, the migrated cells were stained with a fluorescence dye and imaged under the fluorescence microscope (Additional file 1: Figure S2A). Quantitative analysis revealed that the siARID1A- and siARID1A + siACTB-transfected MDCK cells had significantly increased number of the migrated cells as compared with the siControl-transfected MDCK cells (Additional file 1: Figure S2B). However, there was no significant change observed in the siACTB-transfected MDCK cells (Additional file 1: Figure S2B). These data were consistent with the angiogenic data obtained from the ECs incubated with the CM derived from the transfected MDCK cells as described above.

### Effects of single and double knockdowns of *ARID1A *and *ACTB* on chemoresistance of RTECs

Chemoresistance assay was also performed to confirm the carcinogenic features of the *ARID1A*-deficient RTECs. After 24-h incubation with docetaxel, one of the common chemotherapies in cancers, total cell death was quantified to evaluate tolerance of the siRNA-transfected MDCK cells. Flow cytometric analysis revealed that percentage of total cell death significantly reduced in the siARID1A- and siARID1A + siACTB-transfected MDCK cells as compared with the siControl-transfected cells (Additional file 1: Figure S3). However, there was no significant change observed in the siACTB-transfected MDCK cells (Additional file 1: Figure S3). These data were consistent with the RTECs migration results and the angiogenic data obtained from the ECs incubated with the CM derived from the transfected MDCK cells as described above.

## Discussion

In primary structure of ARID1A protein, there are at least two essential functional binding sites, which are conserved among various species [31]. One is the DNA-binding domain (or ARID domain) at N-terminus that is important for chromatin binding of the BAF complex [31, 32]. This ARID domain preferentially binds to double-strand AT-rich sequence in the major groove of DNA [32, 33]. Upon binding of DNA and BAF complex through the ARID domain, the chromatin can be remodelled, resulting in gene transcriptional regulation [34]. Another essential binding site is LXXLL motif that plays role in protein–protein interactions and is found at the C-terminus of the ARID1A structure [31]. In addition to DNA-binding property, ARID1A has been reported to interact with various proteins, including transcription factors [35, 36] and proteins involved in genomic stability [37] and histone deacetylation [10]. Although some of these ARID1A-interacting proteins have been previously identified, their number is relatively small as compared with the interacting partners of other protein complexes.

Herein, we attempted to perform large-scale identification of the ARID1A-interacting proteins by an IP-MS/MS approach using nanoLC-ESI-LTQ-Orbitrap mass spectrometer. Among the 74 unique ARID1A-interacting proteins identified, the well-known interactors of ARID1A in SWI/SNF complex, including BRG1 and SMARCC2 [1, 2], were also detected. The list of the unique ARID1A-interacting proteins in our present study might somewhat differ from that reported previously [37]. Such difference might be due to different renal cells used (MDCK RTECs in this study, but HEK293T embryonic cells in the previous report) [37]. Additionally, different methods for sample preparation and identification should be also taken into account for such difference.

Bioinformatic analysis using STRING tool was done to predict the interactions network of ARID1A and its identified interactors. The network of protein–protein interactions was computed and generated based on the databases obtained from scientific literatures, experimental evidence, computational prediction efforts and systematic transfers of the interaction evidence [38]. It was quite convincing that almost all of these identified ARID1A-interactors showed direct or indirect association with ARID1A. For molecular function annotation, STRING analysis revealed that almost all of the ARID1A interactors exhibited binding function to several molecules. According to these bioinformatic findings, we focused our attention on β-actin, which directly interacted with ARID1A and played role in nucleosome binding. Moreover, recent study has discovered the crucial function of β-actin in gene expression regulation through chromatin remodeling activity [39]. Prior to investigating the function of ARID1A and β-actin, their predicted direct association was validated by IP and reciprocal IP followed by immunoblotting, which are commonly used for validation of the protein–protein interactions [23, 24, 40].

In functional investigations, expression of *ARID1A* and *ACTB* in MDCK renal cells was individually or dually silenced by siRNA technique. Their mRNA and protein levels were decreased approximately 50% by single or double gene siRNA knockdowns. The efficacy of siARID1A knockdown in this study was comparable with that previously done in MDCK and 786-O renal cancer cells [22], Caco-2 colon cancer cells [21], and EA.hy926 ECs [19]. Besides, our data revealed that knockdown of *ARID1A* did not alter mRNA and protein expression levels of β-actin and, on the other hand, silencing *ACTB* showed no effect on ARID1A mRNA and protein levels. As a result, double knockdowns did not add further decrease on each of them. It indicates that their regulation at transcriptional and translational levels are independent from each other, consistent with a recent study demonstrating no correlation between the mRNA expression of *ARID1A* and its interactor, *MSH2* [37].

Deficiency of *ARID1A*, as a tumor suppressor gene, has been thought to significantly affect the transcriptional control of many genes contributing to carcinogenesis in several cancers [36, 37, 41]. As angiogenesis is one of the crucial carcinogenic features in many cancers [42], we thus attempted to investigate the effects of knockdowns of *ARID1A* and its interactor on transcription of angiogenesis-related genes. Angiogenesis involves multiple processes of neovascular formation that is important for nourishing and removing waste products in tumor during its development [43]. At the initial step of a new vascular formation, cancer cells profoundly produce and secrete various angiogenic factors [44, 45]. These angiogenic factors then bind to their specific receptors located on the membrane of ECs [46, 47]. Subsequently, several intracellular signaling cascades are activated to promote ECs proliferation, directional migration to angiogenic signals, and tube formation [44, 48].

After gene knockdowns, expression levels of six angiogenesis-related genes encoding angiogenic factors were determined using semi-quantitative RT-PCR. Our data demonstrated that ARID1A deficiency by both single and double gene knockdowns caused significant increases in *VEGF* and *FGF2* levels. Conversely, *PDGF* and *EGF* levels significantly decreased in the *ARID1A*-deficent cells. Nevertheless, expression of *TGFB1* and *FGF1* was not affected by *ARID1A* and/or *ACTB* knockdowns. Moreover, all of these angiogenesis-related genes were not affected by *ACTB* knockdown alone. These data suggest that ARID1A gene/protein, but not β-actin gene/protein, plays role in regulating the angiogenesis-related genes. In a previous study, VEGF mRNA and protein levels also increased in the *ARID1A*-deficient MCF cells (breast cancer cell line) [20]. Besides, the *ARID1A*-deficient Caco-2 cells showed increased secretion of VEGF [21]. However, deficiency of *Arid1a* in mouse HCC (hepatocellular carcinoma) cells did not alter several of the angiogenesis-related genes, including *Vegfa*, *Fgf2*, *Egfl7*, *Sdf1*, *Hif1a*, *Hif2a* and *Ang1* [18]. These data indicate that the effects of *ARID1A* deficiency or gene knockdown on expression of angiogenesis-related genes may be cell type-dependent. Briefly note that the present study has reported, for the first time, experimental data of the effects of *ARID1A* deficiency on expression levels of *PDGF*, *EGF*, *TGFB1* and *FGF1*.

Previous evidence has demonstrated that up-regulation of genes encoding angiogenic factors correlates with the increased secretion of angiogenic factors from the cells [49–51]. Our present study also addressed this by using ELISA to measure the secreted levels of VEGF and TGF-β1 (the representatives for the up-regulated and unaffected angiogenic factors, respectively). In consistent with those previously reported findings, our present data confirmed that changes in expression of the angiogenesis-related genes really affected the secretion of their protein products. Among the secreted angiogenic factors, VEGF and FGF2 have been reported to trigger ECs activity [50, 51]. Due to the up-regulation of *VEGF* and *FGF2* in our present study, we thus performed in vitro experiments to assess the angiogenesis features induced by CM from MDCK cells with *ARID1A* and/or *ACTB* knockdowns. Our results showed that the migratory activity and tube formation ability of ECs increased after incubation with CM derived from *ARID1A*-deficent MDCK cells. These results were consistent with the study revealing that VEGF detected in CM derived from colorectal cancer cells increased ECs migratory activity and ability to form the tubes [49]. Additionally, VEGF, FGF2 and TGF-β levels in the CM collected from gingival mesenchymal stem cells correlated with the increases of migratory activity and tube formation of ECs [50]. Moreover, a co-culture assay has demonstrated that tumoroids obtained from patients secreted VEGF to induce ECs tube formation [51]. While *VEGF* and *FGF2* increased, *PDGF* and *EGF* had decreased levels, and the other two (*TGFB1* and *FGF1*) had no significant changes in the *ARID1A*-deficent cells in our present study. Interestingly, the overall effect of these contradictory changes of all the six angiogenesis-related genes has shown that the angiogenesis features of ECs exposed to the CM derived from the *ARID1A*-deficent MDCK cells was predominantly determined by the up-regulation of *VEGF* and *FGF2*. These data implicate that *VEGF* and *FGF2* may serve as the more potent angiogenic factors than the others in MDCK cells. However, differential effects of these angiogenic factors may be cell type-specific and should be examined in each of the cells of interest.

Nevertheless, the effect of CM obtained from *ARID1A*-deficient MDCK cells on ECs proliferation was not observed in the present study. It might be explained that ECs proliferation, migration and tube formation are regulated by different signaling pathways [44, 52]. There is evidence demonstrating that the recognition of VEGF by its receptor (VEGFR) can interact with protein kinase C (PKC) for activating RAF/MEK/MAPK pathway, resulting in ECs proliferation [44, 53]. However, this recognition promotes ECs migration through NRP1/FAK [54] and Nck/Fyn/SAPK2/p38MAPK [52, 55] pathways. The interaction of VEGF/VEGFR with NPR1 also stimulates p38 MAPK pathway, leading to the increase of ECs tube formation [56]. FGF2 has been reported to regulate ECs functions by several downstream signaling pathways [57]. These include SRSF1/SRSF3/SRPK1 pathway for promoting ECs proliferation and migration [58] and MAPK-activated AKT/MMP-2 signaling for inducing ECs migration and tube formation [59]. Moreover, FGF2 can increase the level of VEGF [60] and VEGFR [61] and further induces angiogenesis via VEGF-mediated pathways [61].

In addition to angiogenesis, we also confirmed that the *ARID1A* knockdown also enhanced the other carcinogenic features in MDCK RTECs. These carcinogenic features included the increased cell migratory capability and chemoresistance. Our findings demonstrated that *ARID1A* deficiency by single and double knockdowns could enhance the RTECs migratory activity and tolerance ability to a chemotherapy. These findings were consistent with those reported from the previous studies revealing the enhancement of cell migration and chemoresistance in kidney [22] and colon [21] cells after *ARID1A* knockdown. Nevertheless, the *ACTB* knockdown did not affect cell migration and chemoresistance. A recent study has also shown that not only the knockdown but also overexpression of *ACTB* does not alter cell migration [62]. Besides, a more recent study has demonstrated that the level of β-actin in cancer cells is not associated with their chemoresistance [63], consistent with our present study. Possibly, the effects of *ACTB* deficiency might be compensated by the expression of other actin isoforms, which have been reported to get involved in cell migration and chemoresistance [64].

Although the data reported herein are convincing, further extensive investigations are required to strengthen our data and to elucidate the precise physical and functional interactions between ARID1A and β-actin as well as other ARID1A-interacting partners in normal and cancer states. For example, their interactions can be assessed by other several interaction/affinity assays such as yeast-two hybrid system, tandem affinity purification, crosslinking protein interaction analysis, label transfer protein interaction analysis, etc. In addition to the siRNA silencing, restoration of the *ARID1A* expression should be done to solidify the effects from its knockdown. Moreover, analyses will be more meaningful if other cell lines representing RTECs and ECs or their primary cells can be also investigated. Finally, the findings should be confirmed in the in vivo settings or in humans to ensure that they are clinically relevant.

## Conclusions

Our present study has identified a large number of the ARID1A-interacting proteins in RTECs using an IP-MS/MS approach. These identified ARID1A interactors have direct or indirect interactions with ARID1A. Among them, the direct association between ARID1A and β-actin has been confirmed by IP and reciprocal IP followed by immunoblotting. Furthermore, we have investigated their roles in angiogenesis. Functional analyses have shown that siRNA knockdown of *ARID1A*, but not *ACTB*, in MDCK cells significantly increases expression levels of *VEGF* and *FGF2*, but decreases *PDGF* and *EGF*, and has no effect on *TGFB1* and *FGF1* expression. The quantitative mRNA expression data of *VEGF* and *TGFB1* are consistent with the secreted levels of their protein products as measured by ELISA. Because *VEGF* and *FGF2* serve as the potent angiogenic factors, subsequent experiments have revealed that the secreted products derived from the *ARID1A*-deficient MDCK renal cells stimulate migratory activity and tube formation ability of EA.hy926 ECs. Some of the other carcinogenic features can also be confirmed in the *ARID1A*-deficient MDCK renal cells, including the increased cell proliferation and chemoresistance. Taken together, these data indicate that ARID1A interacts with β-actin and several other proteins in MDCK cells. The down-regulation of *ARID1A*, which is a tumor suppressor gene, induces expression of angiogenesis-related genes (particularly *VEGF* and *FGF2*) and stimulates angiogenesis independently of *ACTB* encoding β-actin.

### Supplementary Information

**Additional file 1: Figure S1.** Effects of single and double knockdowns of *ARID1A* and *ACTB* on secretion of angiogenic factors from RTECs. **Figure S2.** Effects of single and double knockdowns of *ARID1A* and *ACTB* on RTECs migration. **Figure S3.** Effects of single and double knockdowns of *ARID1A* and *ACTB* on chemoresistance of RTECs.

**Additional file 2: Supplementary Methods.**

## Data Availability

All data generated or analyzed during this study are included in this published article and are also available from the corresponding author on reasonable request.
