# Supplementary material for: Identification and characterization of ARID1A-interacting proteins in renal tubular cells and their molecular regulation of angiogenesis
Source: J Transl Med. 2023 Nov 28;21:862. doi: 10.1186/s12967-023-04750-y (PMC10683333; doi:10.1186/s12967-023-04750-y)
Supplement: Supplementary file 1 — Additional file 1: Figure S1. Effects of single and double knockdowns of ARID1A and ACTB on secretion of angiogenic factors from RTECs. Figure S2. Effects of single and double knockdowns of ARID1A and ACTB on RTECs migration. Figure S3. Effects of single and double knockdowns of ARID1A and ACTB on chemoresistance of RTECs. [file 12967_2023_4750_MOESM1_ESM.pdf]

ADDITIONAL FILE FIGURES

Identification and characterization of ARID1A-interacting proteins in renal tubular cells and their molecular regulation of angiogenesis

*Sunisa Yoodee, Paleerath Peerapen, Sirikanya Plumworasawat, Thanyalak Malaitad, and Visith Thongboonkerd**

**Correspondence: to thongboonkerd@dr.com (or) vthongbo@yahoo.com*

Additional file Figure S1: Effects of single and double knockdowns of *ARID1A* and *ACTB* on secretion of angiogenic factors from RTECs. The siRNA-transfected MDCK renal cells were cultured in serum-free medium for 24 h. Thereafter, the cleared condition medium (CM) was dialyzed, lyophilized and subjected to ELISA to measure secreted levels of VEGF **(A)** and TGF-β1 **(B)**. The dots on top of each bar represent individual data points derived from three biological replicates in three independent experiments, whereas the error bar represents mean ± SD of each group. Only significant *p* values are labeled.

Additional file Figure S2: Effects of single and double knockdowns of *ARID1A* and *ACTB* on RTECs migration. The siRNA-transfected MDCK renal cells were incubated with serum-free medium in the upper chamber of the Transwell plate and allowed to migrate towards the lower chamber containing complete medium (with serum) for 24 h. Thereafter, the migrated cells were stained with a fluorescence dye and imaged under a fluorescence microscope. **(A)** Micrographs of the immunofluorescence-stained migrated RTECs in each condition. **(B)** Numbers of the migrated RTECs were counted from 15 random fields per each sample. The dots represent individual data points derived from three biological replicates in three independent experiments, whereas the error bar represents mean ± SD of each group. Only significant *p* values are labeled.

Additional file Figure S3: Effects of single and double knockdowns of *ARID1A* and *ACTB* on chemoresistance of RTECs. The siRNA-transfected MDCK renal cells were incubated with 1 µM docetaxel for 24 h. Thereafter, cell death was quantified by flow cytometry. **(A)** Scatter plots of the cells stained with annexin V and/or propidium iodide. **(B)** Percentage of cell death quantified by flow cytometry. The dots represent individual data points derived from three biological replicates in three independent experiments, whereas the error bar represents mean ± SD of each group. Only significant *p* values are labeled.

Fig. S1

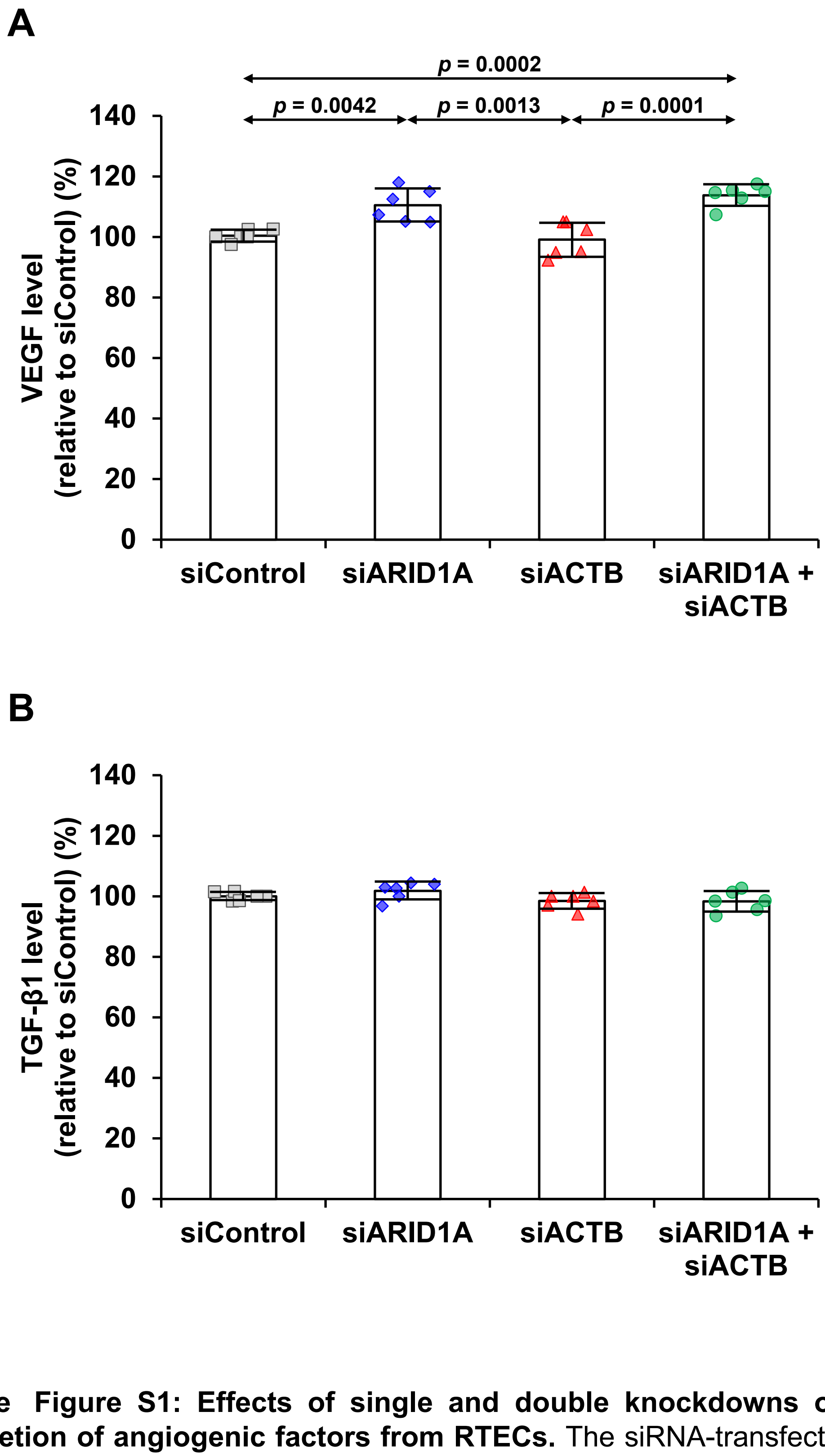

Additional file Figure S1: Effects of single and double knockdowns of *ARID1A* and *ACTB* on secretion of angiogenic factors from RTECs. The siRNA-transfected MDCK renal cells were cultured in serum-free medium for 24 h. Thereafter, the cleared condition medium (CM) was dialyzed, lyophilized and subjected to ELISA to measure secreted levels of VEGF **(A)** and TGF-β1 **(B)**. The dots on top of each bar represent individual data points derived from three biological replicates in three independent experiments, whereas the error bar represents mean ± SD of each group. Only significant *p* values are labeled.

Fig. S2

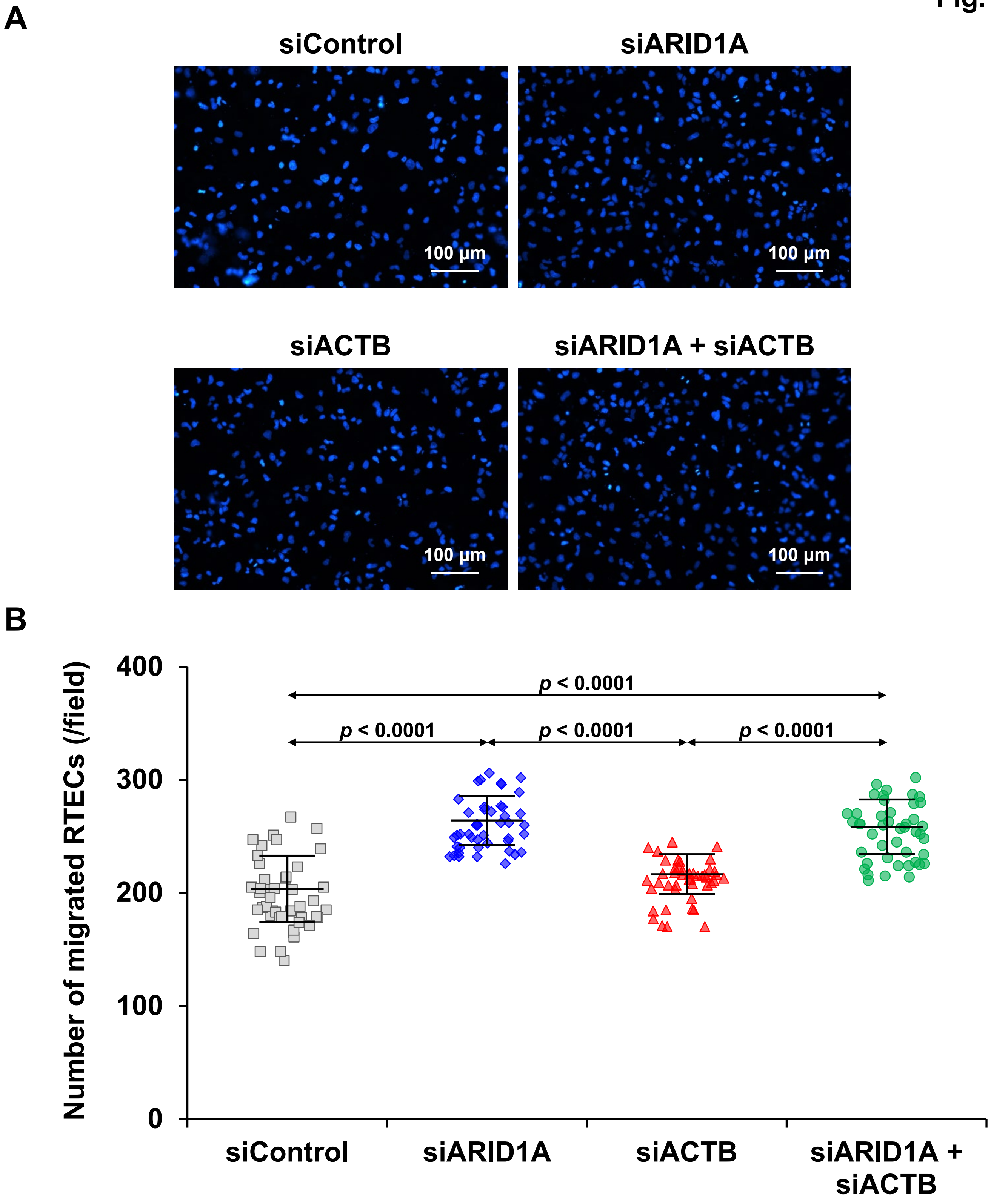

Additional file Figure S2: Effects of single and double knockdowns of *ARID1A* and *ACTB* on RTECs migration. The siRNA-transfected MDCK renal cells were incubated with serum-free medium in the upper chamber of the Transwell plate and allowed to migrate towards the lower chamber containing complete medium (with serum) for 24 h. Thereafter, the migrated cells were stained with a fluorescence dye and imaged under a fluorescence microscope. **(A)** Micrographs of the immunofluorescence-stained migrated RTECs in each condition. **(B)** Numbers of the migrated RTECs were counted from 15 random fields per each sample. The dots represent individual data points derived from three biological replicates in three independent experiments, whereas the error bar represents mean ± SD of each group. Only significant *p* values are labeled.

Fig. S3

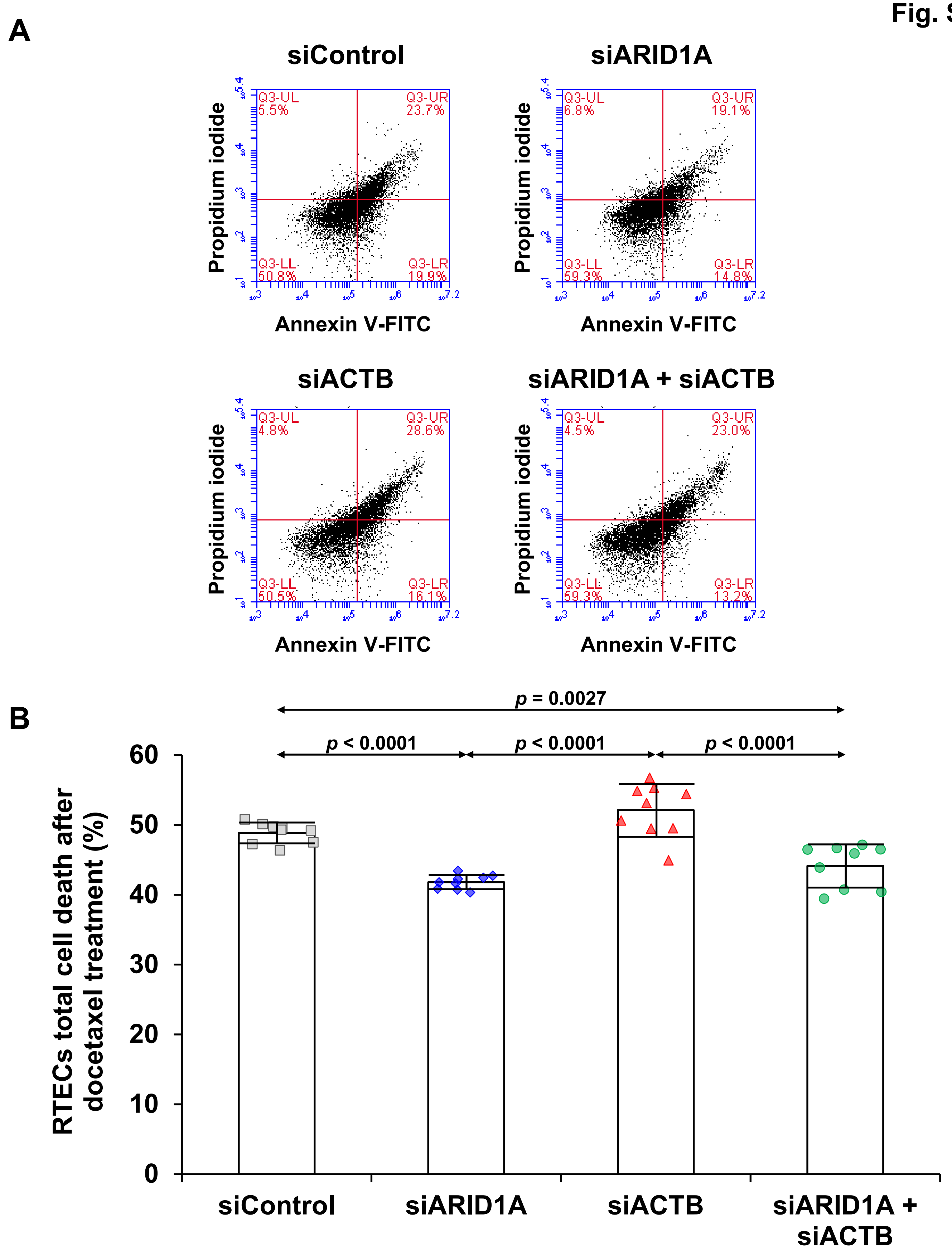

Additional file Figure S3: Effects of single and double knockdowns of *ARID1A* and *ACTB* on chemoresistance of RTECs. The siRNA-transfected MDCK renal cells were incubated with 1 μM docetaxel for 24 h. Thereafter, cell death was quantified by flow cytometry. **(A)** Scatter plots of the cells stained with annexin V and/or propidium iodide. **(B)** Percentage of cell death quantified by flow cytometry. The dots represent individual data points derived from three biological replicates in three independent experiments, whereas the error bar represents mean ± SD of each group. Only significant *p* values are labeled.
