# Supplementary material for: Identification and characterization of ARID1A-interacting proteins in renal tubular cells and their molecular regulation of angiogenesis
Source: J Transl Med. 2023 Nov 28;21:862. doi: 10.1186/s12967-023-04750-y (PMC10683333; doi:10.1186/s12967-023-04750-y)
Supplement: Supplementary file 2 — Additional file 2: Supplementary Methods. [file 12967_2023_4750_MOESM2_ESM.pdf]

ADDTIONAL FILE METHODS

Identification and characterization of ARID1A-interacting proteins in renal tubular cells and their molecular regulation of angiogenesis

Sunisa Yoodee, Paleerath Peerapen, Sirikanya Plumworasawat, Thanyalak Malaitad, and Visith Thongboonkerd*
(*Correspondence to thongboonkerd@dr.com (or) vthongbo@yahoo.com)

In-gel tryptic digestion

Briefly, the gel slices were chopped, washed with 1 ml deionized water, and destained with 100 µl of 100 mM NH_4HCO_3 at 25°C for 15 min. Thereafter, 100 µl acetonitrile (ACN) was added and incubated at 25°C for 15 min. After removing the solvent, the gel pieces were dried in a SpeedVac concentrator (Savant; Holbrook, NY) and rehydrated with 50 µl of 10 mM DTT in 100 mM NH_4HCO_3 at 56°C for 30 min using a heat box. After removing the reducing buffer, the gel pieces were incubated with 50 µl of 55 mM iodoacetamide in 100 mM NH_4HCO_3 at 25°C for 20 min in the dark. The buffer was then removed, whereas the gel pieces were incubated with 100 µl of 50 mM NH_4HCO_3 at 25°C for 15 min. Thereafter, 100 µl ACN was added and incubated at 25°C for 15 min. After removing the solvent, the gel pieces were dried in a SpeedVac concentrator, and incubated with a minimal volume (just to cover gel pieces) of 12 ng/µl sequencing grade modified trypsin (Promega; Madison, WI) in 50 mM NH_4HCO_3 in a ThermoMixer C (Eppendorf; Hauppauge, NY) at 37°C for 16-18 h. The digestion reaction was stopped by incubation with 100 µl of 5% formic acid/ACN (1:2 vol/vol) at 37°C for 15 min. The digested peptide mixtures were collected using a pipette with gel loader tip, transferred into a fresh tube, dried by a SpeedVac concentrator, and subjected to MS/MS analysis.

Analyses of proteins by nanoLC-ESI-LTQ-Orbitrap MS/MS

Separation of the digested peptides was performed using EASY-nLC II (Thermo Scientific; Waltham, MA). Briefly, peptides were loaded from a cooled (7°C) autosampler into an in-house, 3-cm-long pre-column containing 5-µm C18 resin (Dr. Maisch GmbH; Ammerbuch, Germany) (trap column to desalt and concentrate the samples) and then into an in-house, 10-cm-long analytical column packed with 3-µm C18 resin (Dr. Maisch GmbH) using mobile phase A (0.1% formic acid). The peptides were then separated by mobile phase B (ACN/0.1% formic acid) gradient elution with four steps as follows: 2-9% for 4 min, 9-

35% for 30 min, 35-95% for 9 min, and 95% for 5 min at a flow rate of 200 nl/min. Peptide sequences were then analyzed by LTQ-Orbitrap-XL (Thermo Scientific) in positive mode with ESI nanosprayer ion source.

Data were acquired in a collision-induced dissociation (CID) top-12 mode under the control of the Xcalibur 2.1.0 and LTQ Tune Plus 2.5.5 software (Thermo Scientific). The cycle of one full scan was performed at a resolution of 30,000 (300-2,000 m/z) in the Orbitrap followed by 12 data-dependent MS/MS scans in the linear ion trap with enabled preview mode for FTMS master scan. The minimum signal threshold at $1x10^5$ was required for a precursor ion to be selected for further fragmentation. Accumulation target values of full MS and MS/MS scans were $5x10^5$ and $3x10^4$ ions, respectively. Singly charged ions and unassigned charge states were excluded for fragmentation. Helium was used as a collision gas and the normalized collision energy was set at 35%. The activation time was 30 ms for acquiring mass spectra. The duration of dynamic exclusion was 180 s.

MS/MS data processing and protein identification

The raw files (.raw) were analyzed by MaxQuant software package (version 2.1.4.0) (*https://www.maxquant.org*) with built-in Andromeda search engine. Proteins were identified by searching against the UniprotKB/Swiss-Prot mammalian database together with commonly observed contaminants and reversed sequences (decoy database) for all entries. The parameters used for protein identification were set as follows: fixed modification = carbamidomethylation at cysteine (C), variable modification = oxidation at methionine (M), maximal number of missed cleavages = 1, minimum peptide length = 7 amino acids, enzyme = trypsin, first search precursor tolerance = 4 ppm, main search precursor tolerance = 2 ppm, MS/MS tolerance of ion trap mass spectrometer (ITMS) scans = 0.2 Da, and minimum unique peptide = 1. The protein and peptide-to-spectrum match (PSM) false discovery rate (FDR) were set at 0.01 calculated based on the search against reverse sequence decoy database.

Western blotting

Immunoprecipitated proteins or whole cell lysate proteins were denatured by heating at 95°C for 5 min. The proteins were then resolved in 12% SDS-PAGE gel and blotted onto a nitrocellulose membrane using a semi-dry transfer apparatus (GE Healthcare). The membrane was incubated with 5% skim milk/PBS for blocking non-specific bindings and background. The membrane was then probed with mouse monoclonal anti-ARID1A (Santa Cruz

Biotechnology) or anti-β-actin (Santa Cruz Biotechnology) diluted 1:2,000 with 1% skim milk/PBS. After overnight incubation at 4°C, the membrane was further incubated at 25°C for 1 h with rabbit anti-mouse secondary antibody conjugated with horseradish peroxidase (Sigma-Aldrich) diluted 1:20,000 with 1% skim milk/PBS. Immunoreactive protein bands were then visualized by using SuperSignal West Pico Chemiluminescence substrate (Pierce Biotechnology; Rockford, IL).

Single or double gene knockdowns by siRNA in MDCK cells

MDCK cells were grown overnight in antibiotics-free DMEM in each well of the 6-well plate (Corning Costar) to obtain approximately 60-80% confluency. The cells were then incubated with 40 pmol of *ARID1A*-siRNA (Santa Cruz Biotechnology) and/or *ACTB*-siRNA (Santa Cruz Biotechnology) premixed with Lipofectamine 2000 transfection reagent (Invitrogen) in Opti-MEM (Gibco). In other parallel wells, the cells were incubated with an equal amount of a scrambled siRNA (siControl) (Santa Cruz Biotechnology) and served as a negative control. The transfection was incubated in a CO_2 incubator at 37°C for 6 h. Thereafter, the cells were continually incubated in the complete medium for 48 h prior to further functional investigations.

ELISA

After removing cellular debris and particles, 2 ml of the conditioned medium (CM) was collected from the siRNA-transfected MDCK cells, dialyzed against deionized water with several changes, and lyophilized. The dried secretory proteins in the CM were resuspended in 500 µl coating buffer (15 mM Na_2CO_3 and 30 mM $NaHCO_3$; pH 9.4). An equal volume (100 µl) of each sample was then added into each well of the 96-well ELISA plate (Nunc; Roskilde, Denmark) and incubated at 4 °C overnight to immobilize the proteins on the well surface. Thereafter, the sample wells were washed with washing buffer (0.05% Tween-20 in PBS) three times and incubated with 1% BSA/PBS at 25 °C for 2 h to prevent non-specific bindings. After other three washes, the samples were incubated with mouse monoclonal anti-VEGF (Santa Cruz Biotechnology) or anti-TGF-β1 (Santa Cruz Biotechnology) (1:50 in 0.1% BSA/PBS) at 37 °C for 2 h. After the other three washes, the samples were further incubated in the dark with rabbit anti-mouse secondary antibody conjugated with horseradish peroxidase (Sigma-Aldrich) (1:5,000 in 0.1% BSA/PBS) at 37 °C for 2 h. After the other three washes, 100 µl chromogenic substrate (3.3 mM *o*-phenylenediamine dihydrochloride, 35 mM $C_6H_8O_7{\cdot}H_2O$, 100 mM $Na_2HPO_4{\cdot}12H_2O$, 0.06%

H_2O_2; pH 5.0) was added into each well and incubated in the dark at 25 °C to develop the reaction color. Finally, 50 µl of 2 M H_2SO_4 was added to stop the reaction, and the absorbance of each sample was measured at wavelength at λ492 nm using an ELISA microplate reader (EZRead 400, Biochrom Ltd.; Cambridge, UK).
